# Ubiquitous Crossmodal Stochastic Resonance in Humans: Auditory Noise Facilitates Tactile, Visual and Proprioceptive Sensations

**DOI:** 10.1371/journal.pone.0002860

**Published:** 2008-08-06

**Authors:** Eduardo Lugo, Rafael Doti, Jocelyn Faubert

**Affiliations:** Visual Psychophysics and Perception Laboratory, School of Optometry, University of Montreal, Montreal, Quebec, Canada; The Rockefeller University, United States of America

## Abstract

**Background:**

Stochastic resonance is a nonlinear phenomenon whereby the addition of noise can improve the detection of weak stimuli. An optimal amount of added noise results in the maximum enhancement, whereas further increases in noise intensity only degrade detection or information content. The phenomenon does not occur in linear systems, where the addition of noise to either the system or the stimulus only degrades the signal quality. Stochastic Resonance (SR) has been extensively studied in different physical systems. It has been extended to human sensory systems where it can be classified as unimodal, central, behavioral and recently crossmodal. However what has not been explored is the extension of this crossmodal SR in humans. For instance, if under the same auditory noise conditions the crossmodal SR persists among different sensory systems.

**Methodology/Principal Findings:**

Using physiological and psychophysical techniques we demonstrate that the same auditory noise can enhance the sensitivity of tactile, visual and propioceptive system responses to weak signals. Specifically, we show that the effective auditory noise significantly increased tactile sensations of the finger, decreased luminance and contrast visual thresholds and significantly changed EMG recordings of the leg muscles during posture maintenance.

**Conclusions/Significance:**

We conclude that crossmodal SR is a ubiquitous phenomenon in humans that can be interpreted within an energy and frequency model of multisensory neurons spontaneous activity. Initially the energy and frequency content of the multisensory neurons' activity (supplied by the weak signals) is not enough to be detected but when the auditory noise enters the brain, it generates a general activation among multisensory neurons of different regions, modifying their original activity. The result is an integrated activation that promotes sensitivity transitions and the signals are then perceived. A physiologically plausible model for crossmodal stochastic resonance is presented.

## Introduction

Stochastic resonance (SR) [1] is a nonlinear phenomenon whereby the addition of noise can improve the detection of weak stimuli. An optimal amount of added noise results in the maximum enhancement, whereas further increases in noise intensity only degrade detection or information content. The phenomenon does not occur in linear systems, where the addition of noise to either the system or the stimulus only degrades the measures of signal quality. The SR phenomenon was thought to exist only in stochastic, nonlinear, dynamical systems but it also exists in another form referred to as ‘threshold SR’ or ‘non-dynamical SR’. This form of stochastic resonance results from the concurrence of a threshold, a subthreshold stimulus, and noise. These ingredients are omnipresent in nature as well as in a variety of man-made systems, which accounts for the observation of SR in many fields and conditions. The SR signature is that the signal-to-noise ratio, which is proportional to the system's sensitivity, is an inverted U-like function of different noise levels. That is, the signal-to-noise ratio first is enhanced by the noise up to a maximum and then lessened. The SR phenomenon has been shown to occur in different macro [2], micro[3] and nano physical systems [4]. From the cyclic recurrence of ice ages, bistable ring lasers, electronic circuits, superconducting quantum interference devices (SQUIDs) and neurophysiological systems [5] such as receptors in animals. Several studies have suggested that the higher central nervous system might utilize the noise to enhance sensory information [1]. SR studies in humans can be divided in unimodal SR (signal and noise enter the same sense) [6], [7], central SR (signal and noise enters in similar local receptors and later mix in the cortex) [8] and behavioral SR (similar to central SR but its effect is observed in one sense and then enacted in the behavior of the subjects) [9]. Before the SR principle was proposed, Harper [10] discovered what we currently would call crossmodal stochastic resonance while studying the effect of auditory white noise on sensitivity to visual flicker. Recently a similar result [11] has been found where auditory noise produces SR when subthreshold luminance stimuli are present. However what has not been explored is the extension of these interactions in humans. New results show that the noise induces large scale phase synchronization of human-brain activity associated with behavioral SR [12]. It is shown that both detection of weak visual signals to the right eye and phase synchronization of electroencephalogram (EEG) signals from widely separated areas of the human brain are increased by addition of weak visual noise to the left eye. These results imply that noise-induced large-scale neural synchronization may play a significant role in information transmission in the brain. Interestingly SR can be seen as a synchronization-like phenomenon between two energy states of a physical system for example [13]. Furthermore, the synchronization-like phenomenon plays a key role in the enhancement of the signal-to-noise ratio in SR. Another recent result shows that certain multisensory integration interactions (between auditory, visual and somatosensory systems) present similar SR dynamics and the synchronization is not only central but it extends to peripheral systems [14]. Therefore, we can hypothesize that if the noise induces large-scale phase synchronization in different areas of the cortex and peripheral systems with dynamics similar to SR, the crossmodal SR would be a ubiquitous phenomenon in humans because it involves different cortical areas and peripheral systems. Consequently in this work we investigate if, under the same auditory noise conditions, the crossmodal SR is present among tactile, visual and proprioceptive sensory systems. Furthermore, in previous work [10], [11] only visual stimuli classified as first order stimuli were used. We wanted to evaluate the effect of SR on an additional visual attribute called second order processing. For first order stimuli, the local spatial average luminance varies throughout the stimulus while the local contrast remains constant. In second order stimuli, known to be processed by separate mechanisms and assumed to be more complex to process, the local spatial average luminance remains constant but the local contrast varies throughout the stimulus [15], [16]. In summary, we have introduced auditory noise and tested tactile, visual and proprioceptive sensations in humans. We show in a first series of experiments that this will improve tactile sensitivity according to SR theory. In a second series of experiments the SR effects on the visual system were studied by using a more standard luminance-defined first order stimulus than the ones used in [10], [11]. In a third series of experiments the SR effects on the visual system were explored in more detail by going beyond first order visual properties as in [10], [11] but also assess the more complex contrast-defined second-order stimuli [15], [16]. The data demonstrate that SR is present in both types of visual processing. In the last series of experiments we show that the same type of noise can also alter EMG signals during postural control. Our study unveils that crossmodal SR is well extended in humans showing that this phenomenon seems to be a ubiquitous property.

## Results

### General

The study received ethical approval from the Institutional Review Board of the University of Montreal, Quebec, Canada. We performed physiological and psychophysical measurements in a sample of 21 healthy subjects (25–52 years old) with no history of auditory, tactile, visual and motor disorders or detectable neurological disorders. Vision was normal or corrected to normal and we tested hearing in all subjects, which was within normal range for everyone (between 250–8000 Hz). In all the experiments we applied different auditory noise intensity levels plus a baseline (no auditory noise) in randomized order. This randomized order of sessions assured that the observed effects are not simply due to a generalized modulation in attention/arousal. We maintained the intensity of the continuous auditory input noise constant for each session and varied it between sessions. We measured an intensity of 50±3.5 dBSPL with a cut-off frequency of 2.5 kHz for the baseline condition by using a calibrated microphone (see methods).The baseline condition sound pressure level (SPL) is due to the testing room sound disturbances (e.g., computer fans and low power sounds coming from outside the testing room). The auditory noise we used had a cut-off frequency of about 12 to 15 kHz (the original white noise spectrum is attenuated due to the different processing stages required to reach the cortex).We have measured absolute tactile (in microns) and visual (in arbitrary units) thresholds and the absolute EMG (electromyography) activity (in Volts) for posture. The power spectral density (PSD) for the EMG measurements was calculated and the power was obtained by integrating the PSD in the frequency domain (from 0 to 500 Hz). Normalized thresholds and power were computed as follows: once the absolute threshold (or power) was obtained for different auditory noise conditions, their values were divided by the absolute threshold (or power) measured for the baseline condition. Wilcoxon tests were performed to measure if the noise and control conditions were significantly different (p<0.05). In all the graphs error bars represent one standard error. We used two criteria to decide where the SR peak was located in every subject. First, the SR peak was the peak that had the absolute minimum value even if the u-shape function was not fully developed in the noise interval used and second, the peaks had to have a p<0.05. If the subject's peaks did not fulfill the afore-mentioned criteria they were not taken into account in the analysis.

### SR interactions between auditory noise and tactile signals

**Figure 1:**
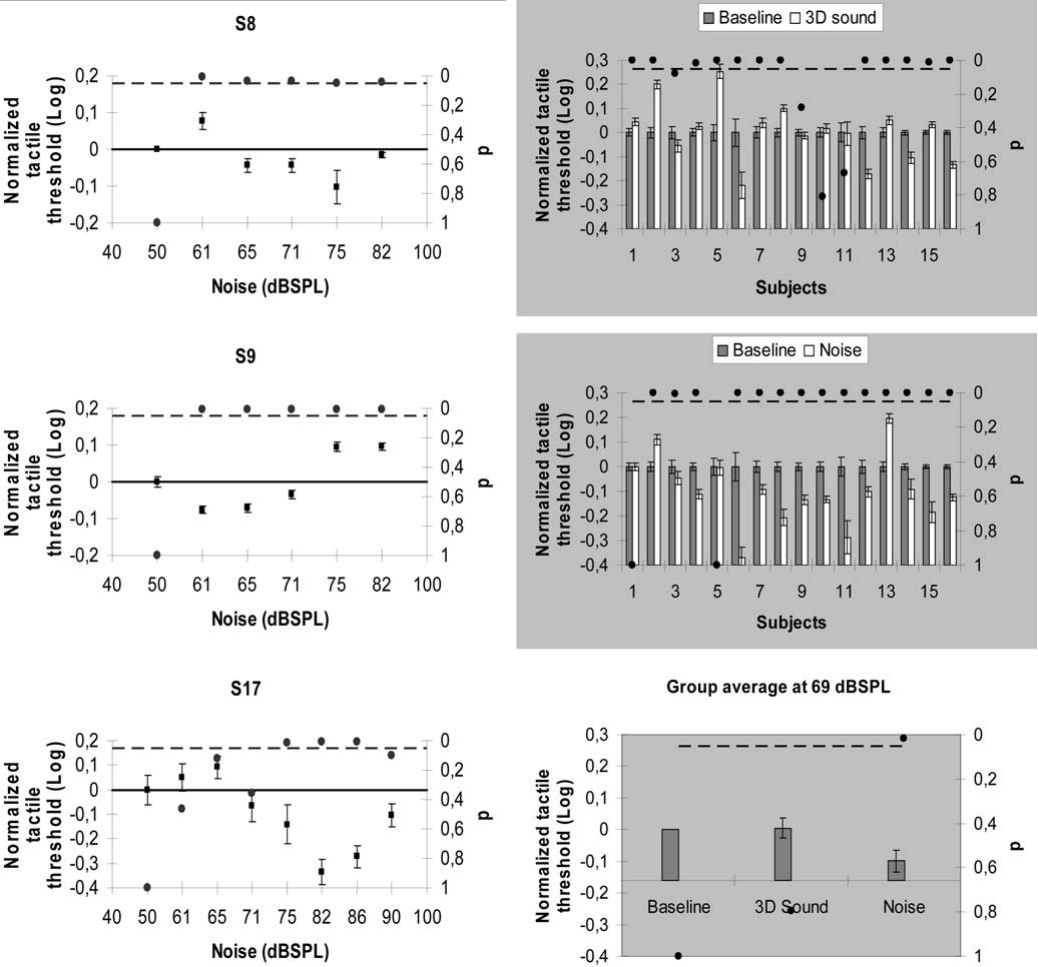
SR interactions between auditory noise and tactile signals. (Left column) normalized tactile threshold changes with the noise level in three subjects. (Right column, top) normalized tactile thresholds of sixteen subjects when the 3D sound level was fixed at 69 dBSPL. (Right column, middle) normalized tactile thresholds of sixteen subjects when the noise level was fixed at 69 dBSPL. (Right column, bottom) Group average results for three conditions: baseline, 3D sound and noise. The average group threshold decreased significantly in the presence of noise (p<0.001) and no significant change was found for the 3D-like sound (p = 0.72). In all the graphs the no-noise condition is taken as baseline; the black dots indicate p-values (right y-axis) and the broken line represents the 5% significance level. Error bars correspond to one standard error.

In the first series of experiments we studied the effects of auditory noise on tactile sensations in three subjects. Tactile vibrations were delivered to the middle finger of the right hand of the subjects at a frequency of 100 Hz and were asked to report the tactile sensation. If they felt the signal they had to click on a yes button or on a no button otherwise. It is known that the yes-no procedure is not free of subject's criterion effects [17] and this may be a limitation of the MEDOC system that we used (see methods). However the subject's criteria can be manipulated and controlled through instructions [18]. For this reason and to better control the subject's decision criteria we did a couple of manipulations. First we asked all the subjects to focus more on the hits (the tactile stimulus is present and the subject responds yes) than on correct rejections (the tactile stimulus is absent and the subject responds no) as it has been shown that this reduces criteria-related effects [19]. Second, response biases or false alarms (the tactile stimulus is not present and the subject responds yes) were controlled in a stringent fashion by repeating the entire sequence from the moment a false alarm occurred and the moment where the previous catch trial was correctly identified (correct rejection). Each subject was tested twice for every auditory noise and baseline condition. Figure 1 (left column) shows the normalized tactile thresholds for three subjects and it is clear that, as the noise level increased, the threshold decreased reaching a minimum and then increased in a typical SR signature fashion. In general we found that the subject's minimum peaks are not always localized at a specific noise level but within a band centered at 69±7 dBSPL.

### Noise effects versus attention/arousal effects on tactile sensations

Can the above results be explained only on the bases of SR theory? Can one potentially rule out an explanation based on attention/arousal? If the noise creates a more interesting/arousing condition than the baseline condition, all neural systems could be correspondingly more excitable, not because the noise facilitates a resonance like behaviour but because the auditory noise non-specifically boosts neural excitability. However, the Yerkes-Dodson law demonstrates an empirical relationship between arousal and performance [20]. Such relationship is task dependent. For instance, in a simple task the relationship between arousal and performance is linear and only in a difficult task this relationship becomes curvilinear (inverted u-shape similar to SR). Since a yes-no procedure with vibration thresholds would be considered a very simple task, we would not expect an inverted u-shape between the noise level and tactile sensitivity if the mechanism involved in these interactions was only arousal. That was not the case as Fig. 1 clearly shows a curvilinear relationship. In order to further explore the notion of possible attention effects we performed an additional experiment on sixteen subjects where we used two different auditory stimuli plus the baseline condition. One stimulus was a specific auditory noise condition as described above, and another was a 3D-like sound. Both sounds had an intensity of 69 dBSPL and the 3D sound contained frequencies in a similar range as the auditory noise (between 100 Hz up to 19 kHz). The 3D sound gave the impression of very close movements near, up and down, and around the subjects' head resulting in a very strong attention getting sound sequence. If our previous results were only a result of attention modulation created by the sound intensity, we should expect that for, the 3D auditory condition, the tactile thresholds would be lower in most people because this sequence had strong attention modulation properties and the noise level we chose was the same as the averaged peak noise level we measured in the first experiment that generated the lowest tactile thresholds. An alternative hypothesis is that this attention-producing stimulus would not influence or maybe even hinder tactile performance. On the other hand, we did expect the auditory noise condition to generate lower tactile thresholds given that we chose the averaged peak noise level that generated the lowest thresholds in the previous experiment. Each subject was tested twice for every condition in randomized order. Fig. 1 (right column, top) shows the normalized tactile thresholds for the 3D sound and baseline conditions. Eight subjects augmented significantly their thresholds comparatively to baseline condition, four subjects lessened theirs thresholds and in other four subjects the threshold values remained unchanged. Fig. 1 (right column, middle) shows the normalized tactile thresholds for the auditory noise and baseline condition. Twelve subjects significantly lessened their thresholds, only two subjects increased their thresholds and another two subjects had unchanged threshold values. Fig. 1 (right column, bottom) shows the group average of the normalized tactile threshold for the three conditions. The average group sensitivity increased significantly (with respect the baseline) in the presence of noise (p<0.001) while no significant change was found for the 3D-like sound (p = 0.72). It is clear from these experimental controls, that the noise effects on tactile sensations are not due to attention/arousal effects but result from the way the brain processes the energy (and probably the frequency) content of noise and signal.

### SR interactions between auditory noise and first order visual signals

**Figure 2:**
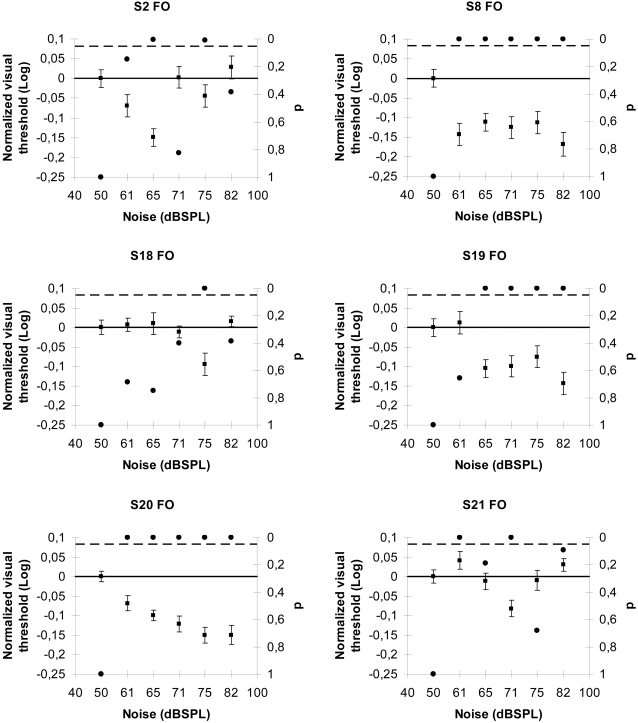
SR interactions between auditory noise and first order visual signals. Normalized visual threshold changes with the noise level in sixth subjects for luminance modulated (first order) stimuli. In all the graphs the no-noise condition is taken as baseline; the black dots indicate p-values (right y-axis) and the broken line represents the 5% significance level. Error bars correspond to one standard error.

In the second series of experiments, we studied whether auditory noise can facilitate luminance-modulated (first order) stimuli detection in six subjects. To evaluate visual thresholds, we used a two-alternative forced choice paradigm (see methods). In a two-alternative forced choice paradigm, the subject is presented two choices and must pick one (even if the observer thinks he/she did not see the stimulus), which produces a more stringent control of observer criteria than a yes/no response. Here the observers had to discriminate between vertical or horizontal luminance-modulated stimuli (LM) defined sinusoidal gratings [15], [16]. We measured the LM thresholds for six auditory conditions (baseline plus five noise levels) in a random order. Five thresholds (5 separate staircases) were established for each condition and averaged. Fig. 2 shows the normalized visual LM thresholds for six subjects. As in our previous auditory-tactile experiments, the visual threshold profiles of the observers varied as a function of the different auditory noise levels demonstrating a typical SR function with zones of threshold values significantly different from the control condition. The SR average peak for our data was 75±3 dBSPL for LM stimuli. Previous reports show an average value of 70±2.5 dBSPL for visual flicker detection [10] and a value of 73.8±15.5 dBSPL for a luminance-defined stimulus [11].

### SR interactions between auditory noise and second order visual signals

**Figure 3:**
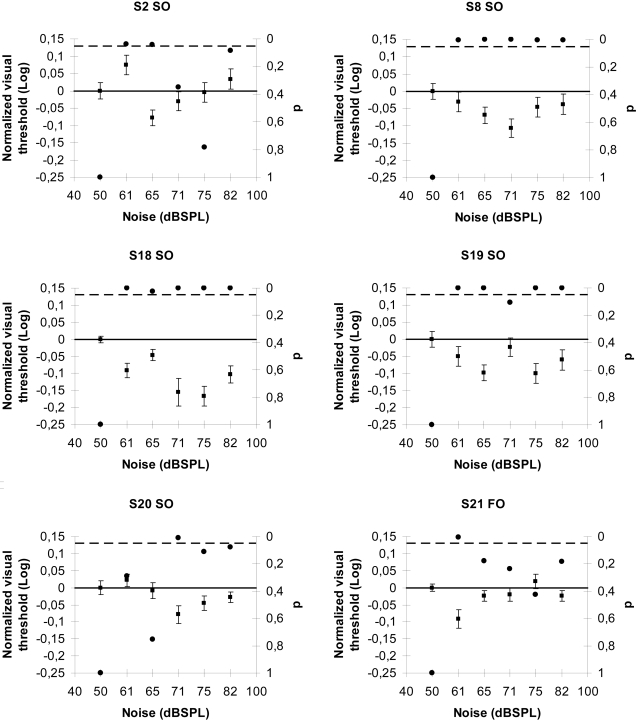
SR interactions between auditory noise and second order visual signals. Normalized visual threshold changes with the noise level in sixth subjects for contrast modulated (second order) stimuli. In all the graphs the no-noise condition is taken as baseline; the black dots indicate p-values (right y-axis) and the broken line represents the 5% significance level. Error bars correspond to one standard error.

In the third series of experiments, we studied whether auditory noise can facilitate contrast-modulated (second order) stimuli detection. With the same procedure as above, the observers had to discriminate between vertical or horizontal contrast-modulated stimuli (CM) defined sinusoidal gratings [15], [16]. We measured the CM thresholds for six auditory conditions (baseline plus five noise levels) in a random order. Five thresholds (5 separate staircases) were established for each condition and averaged. Fig. 3 shows examples of the normalized visual CM thresholds for the same six subjects. As in our previous auditory-visual experiments, the visual CM threshold profiles of the observers varied as a function of the different auditory noise levels demonstrating a typical SR function with zones of threshold values significantly different from control. The SR average peak was found at 70±2 dBSPL for CM stimuli. Clearly both peaks are inside the same experimental region and there is no significant difference between them meaning that within the experimental accuracy we have used both SR mechanisms are similar.

### SR interactions between auditory noise and propioceptive signals

**Figure 4:**
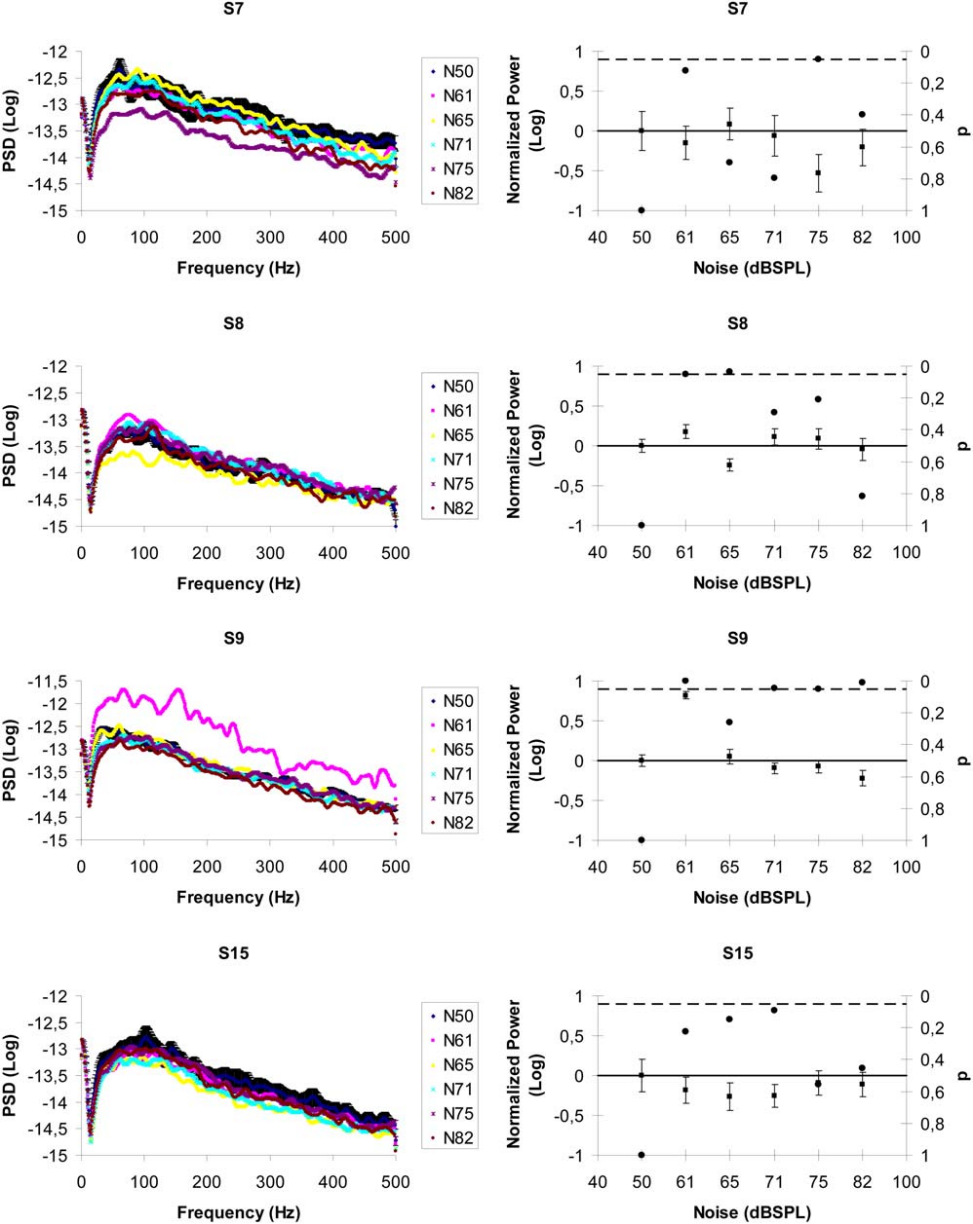
SR interactions between auditory noise and propioceptive signals. (Left column) average EMG power spectral densities as a function of noise level in four subjects for the tightrope posture position. For clarity only the baseline condition shows error bars (one standard error). (Right column) normalized power in four subjects. Again, the no-noise condition is taken as baseline; the black dots indicate p-values (right y-axis) and the broken line represents the 5% significance level. Error bars correspond to one standard error.

In the last series of experiments we evaluated electromyography (EMG) responses of the subjects' leg muscles during posture maintenance with different auditory noise conditions. Recent evidence has demonstrated that tactile stimulation of the foot with noise could increase postural stability by acting on the somatosensory system and that noise can induce transitions in human postural sway [21]–[23]. Four subjects were asked to stand with their feet aligned one in front of the other and touching like in a tightrope position. For all conditions (the baseline plus five noise levels) we have measured the EMG activity (amplification gain of 1000 and sampling rate of 1000 Hz) of each subject three times in a randomized order. In figure 4 (left column) we show the averaged EMG power spectrum density as a function of noise intensity in four subjects. The right column of figure 4 shows the normalized power of the EMG activity in the same four subjects with different noise levels and the baseline. The EMG activity refers to the muscle's activity during posture maintenance. In this context a less stable posture represents more activity of the muscles related to this task. Again the SR signature was observed by using similar noise levels as the tactile and visual experiments and surprisingly, the subjects' averaged peak 74±4 dBSPL lies in the same experimental range found in our previous experiments.

### General model for crossmodal SR

In this section we present a biologically plausible model that can accommodate the notion of crossmodal SR and our present results. We can simulate neurons as natural devices with dynamics that consist of random low-amplitude motions (spontaneous neuronal activity) from which escapes occur at certain intervals [24]. The escapes are referred to as firings, and are associated with high amplitude bursts (spikes). An optimal model should also fulfill the following conditions:

1. Be both energy and frequency based (the excitation energy could be stochastic or deterministic).
2. Reproduce the spontaneous activity of neurons.

We begin by proposing a similar bistable model for the response of neurons as in [24]

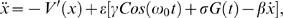
(1)Where *x* represents the neurons' amplitude activity, *x* ˙ is the neurons' amplitude activity velocity (how their activity changes with time), *V*(*x*) is a double-well potential defined by a polynomial, *ε* is a perturbation parameter that may have a stepwise variation over *x*. *G*(*t*) is a nearly white noise process, *γ*, *σ* and *β* are adjustable parameters. Additional model parameters are those defining the polynomial *V*(*x*), and the constants defining the stepwise variation of *ε*. We note that the dimensional counterparts of the terms *x* ¨, *εγCos*(2*πf*_0_ *t*), and (*ω*_0_ = 2*πf*_0_), *εσG*(*t*) are respectively *d* ^2^ *Y*/*dτ* ^2^, *εA*_0_ *P*_0_ *Cos*(2*πF*_0_ *t*), and *εAPG*(*t*) where *Y* = *c*_1_ *x*, *τ* = *c*_2_ *t*, *F*_0_ = *f*_0_/*c*_2_, *Y*, *τ*, *A*_0_ and *A* have dimensions of *mV*, *ms* and *mV*/*ms* ^2^, respectively, with *P*_0_ and *P* expressed in dB. Thus 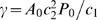, and 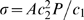. Typical amplitudes of firings in the auditory nerve are about 1 mV.

**Figure 5:**
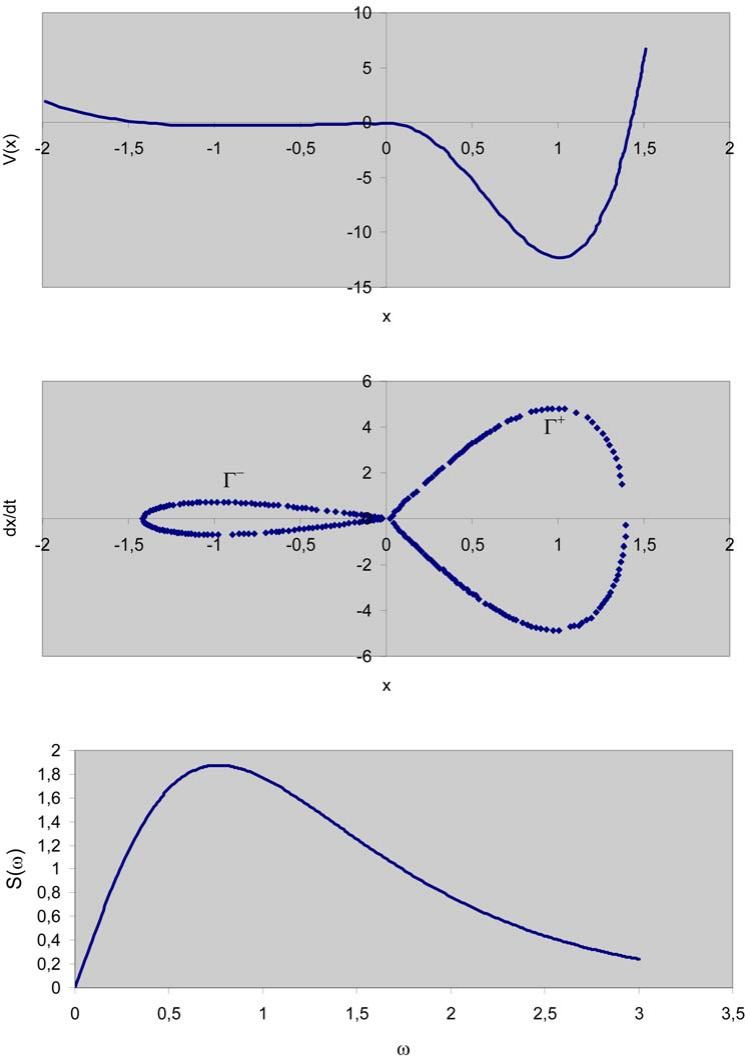
Theoretical model for crossmodal SR. (Top) Potential V(x); (middle) Phase plane diagram showing homoclinic orbits; (bottom) Melnikov scale factor for *α* ^−^ = 1.

Equation (1) can achieve simulations of neuronal time histories (with the appropriate parameter values) and it has solutions with the qualitative features observed in SR described earlier. To achieve good neuronal time history simulations, the potential *V*(*x*) must be asymmetric, which is deeper for *x*>0 than for *x*≤0 as shown in figure 5 (top).

#### Neuronal firing necessary condition

Associated with an unperturbed system (*ε* = 0 for all *x*) are the homoclinic orbits Γ^+^ and Γ^−^ shown in figure 5 (middle). In order for the escapes to take place we require that the maximum total energy produced during the motion over an entire homoclinic loop will be bigger than zero. Suppose the motion takes place on the unperturbed system's homoclinic orbit. If the motion occurs over a small distance *δx_h_* (*h* designates coordinates of the homoclinic orbit), then the energy loss *δ_loss_* during this motion is equal to the damping force *δx_h_*, that is,
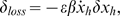
(2)and the total energy losses during this motion over the entire homoclinic orbit are
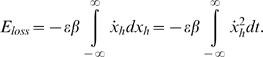
(3)The energy gained during a motion over the entire homoclinic orbit loop is equal to the excitation energy, that is,
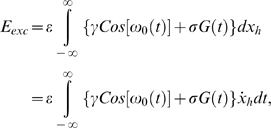
(4)The energy contributed by the potential force V(x) during a motion over the entire homoclinic loop is zero. The total energy produced during the motion over an entire homoclinic loop is therefore:
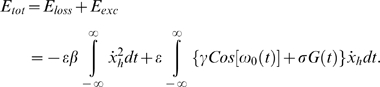
(5)The condition max(*E_tot_*)>0 implies that the maximum of the second term between braces in equation (5) is larger than the first term. Equation (5) implies that the energy of the system can drive the motion over the potential barrier and out of a potential well. Note that with no loss of generality, we can choose *ε* = 1. This merely affects the choice of the parameters *γ*, *σ* and *β*. For any specified excitation *ε*[*γCos*(*ω*_0_ *t*)+*σG*(*t*)], *εβ* must therefore be chosen to warranty that equation (5) is bigger than zero. The fact that condition (5) is fulfilled allows the motion to escape from the inner core (figure 5, middle), that is, the motion can follow a trajectory inside or outside the homoclinic orbit Γ^−^. After reaching the coordinate *x* = 0 the motion continues in the half-plane *x*>0. Lets assume that in this half plane the perturbation vanishes (*ε* = 0) or is very small (*ε*≪1) the motion cannot cross into the inner core defined by the homoclinic orbit Γ^+^. Rather, a large-amplitude motion close to Γ^+^ occurs that returns the trajectory to the half-plane *x*≤0, where it again stays outside or it is entrained into the inner core, meaning that in this region each trajectory that intersects the axis *x* ˙ does so at a different point, so that no two trajectories near Γ^+^ are the same.

#### The potential *V*(*x*)

For a fixed maximum homoclinic orbit coordinate *x*_max_≠0 such that *V*(*x*_max_) = 0, the deeper a potential well the larger are the velocities in trajectories close to its homoclinic orbit (the velocity being equal to the ordinate of the trajectory in the phase plane). The choice of the depth of the well for the half-plane *x*>0 is dictated by the need to achieve a relatively small time of travel for the motions in that half-plane (neuron firing simulation). It is reasonable, at least to a first approximation, to try the potential:
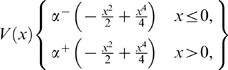
(6)Which represents an asymmetric, modified version, of the Duffing-Holmes potential with a saddle point at *x* = 0. The coordinate *x*_max_ is independent of *α* ^+^. On the other hand, the larger *α* ^+^, the deeper is the well and the larger are the velocities on and near the homoclinic orbit.

#### The homoclinic orbits

In the phase space defined by (*x_h_*, *x* ˙*_h_*), the orbits connecting the saddle point to itself are called homoclinic. They are orbits with infinitely long periods. Such orbits are sometimes referred to as nongeneric and are defined by the energy condition:
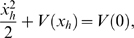
(7)Which gives the initial conditions 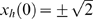 and *x* ˙*_h_*(0) = 0, where the plus and minus signs denote the orbit in the positive and negative *x* half planes, respectively. Now by using the system's Halmitonian function that physically represents the total energy:
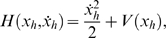
(8)and since we know the initial conditions (*x_h_*(0), *x* ˙*_h_*(0)) for the homoclinic orbits thus *H*_0_ = *H*(*x_h_*(0), *x* ˙*_h_*(0)) can be known and (*x_h_*, *x* ˙*_h_*) are obtained from:
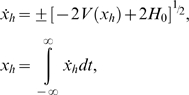
(9)Hence, *x_h_* and *x* ˙*_h_* are given by the expressions:
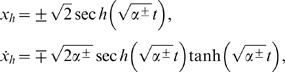
(10)


### Approximate Bennett-Rice representation of a nearly white stochastic process

A good alternative representation of the *G*(*t*) process with zero mean, unit variance, and one-sided spectral density Ψ_0_(*ω*) is the Bennett-Rice representation
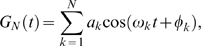
(11)where *a_k_* = [Ψ_0_(*ω_k_*)Δ*ω*/(2*π*)]^½^, *φ_k_* are uniformly distributed over the interval [0,2*π*], *ω_k_* = *k*Δ*ω*, Δ*ω* = *ω_cut_*/*N*, and *ω_cut_* is the frequency beyond which Ψ_0_(*ω*) vanishes or becomes negligible (cutoff frequency). The one-sided spectral density is given by
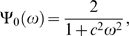
(12)In which *c* needs to be small to assure that the spectral density varies slowly with frequency and therefore equation (11) is a close approximation of white noise.

#### Mean escape rate and SR

The harmonic excitation in equation (1) is assumed to have small enough amplitude that by itself (no stochastic excitation) it is unable to transfer the activity from one well to another. However, under the combined action of the harmonic excitation and the stochastic excitation, escapes do occur. We denote the mean escape rate from a well under that combined action, by *α*. For zero noise excitation, *α* = 0, for very small noise excitation *α*<*ω*_0_, but as the noise excitation increases and *α*≈*ω*_0_, there occurs a synchronization-like phenomenon (cooperative effect) that results in an enhancement of the signal to noise ratio. The mean escape rate can be estimated from the mean escape time [24] that for a white noise process *εσG*(*t*) is given by
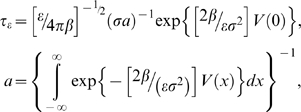
(13)Where *β* is the loss constant of equation (1).

#### Unimodal SR neuron's firing condition

**Figure 6:**
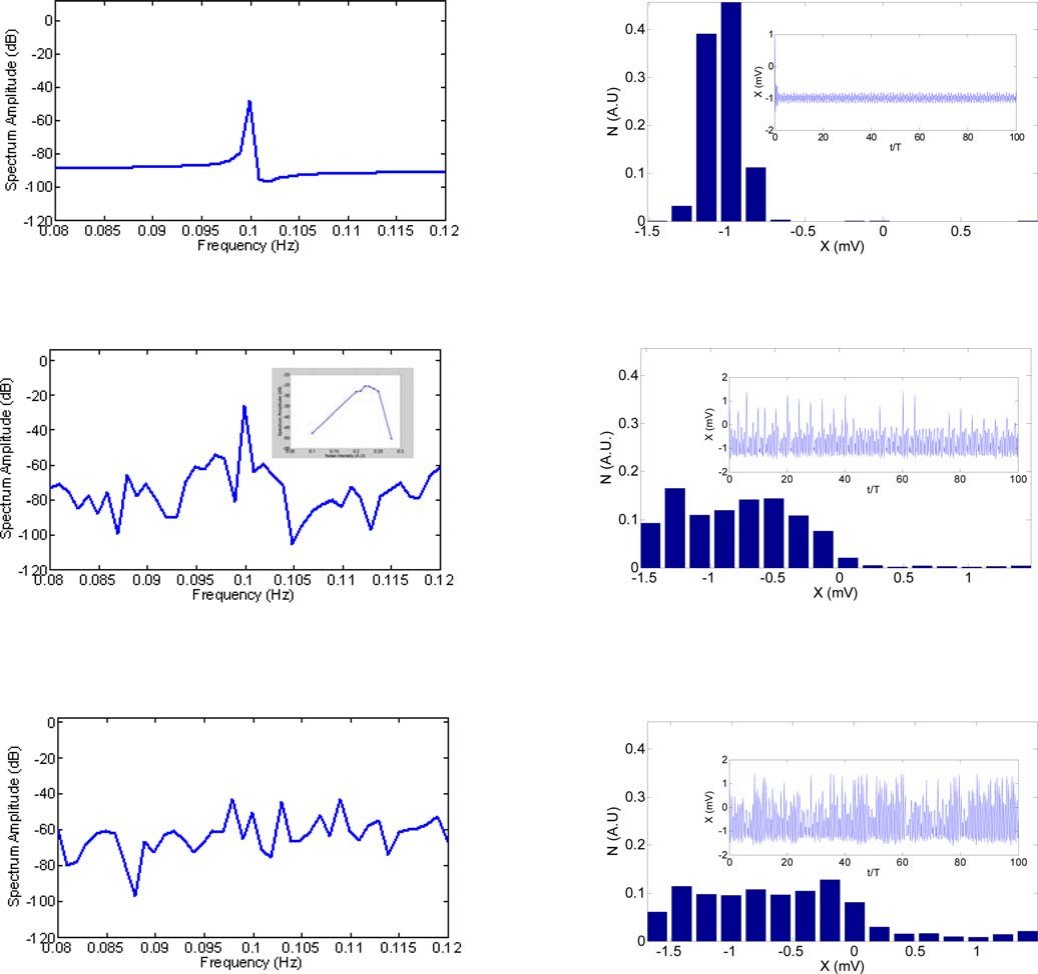
Theoretical model results for unimodal SR. (Left column) shows the neurons' spectrum amplitude as a function of the noise intensity *σ* ^−^. The insert (left column, middle row) shows the well-known SR inverted u-shape function. The maximum peak is found when *P* = 10 *dB*. Right column shows neuronal firing histograms with their corresponding time histories. T is the signal period and N means the probability to have certain neuronal activity levels.

For unimodal SR neurons, the signal (harmonic term in equation 1) and the noise are present in the same region *x*≤0. In this case the inherent system perturbation is defined by parameter values *ε* ^−^>0 for *x*≤0 and *ε* ^+^ = 0 for *x*>0. Hence, in order to calculate the neuron's firing necessary condition, we only have to take in account the homoclinic orbit Γ^−^ and parameters associated with this region. Substituting equation (11) and equation (10), for Γ^−^, into equation (5) and then working out the integrals, the necessary condition for the escapes to take place is
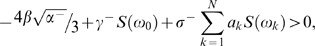
(14)where 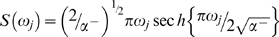 is known as the Melnikov scale factor, shown in figure 5 (bottom) where is clear that if we want to optimize the energy transfer from the stochastic process *G*(*t*) then its spectral density needs to contain frequencies around the Melnikov scale factor maximum. The necessary escape condition (equation 14) serves to calculate the required noise amplitude *σ* ^−^, for neuron firings and the mean escape rate equation gives us the condition to observe the SR peak in neurons. Figure 6 shows some simulations for the behavior of neuronal activity with different noise amplitudes. The parameters we used are *ε* ^−^ = 1 *γ* ^−^ = 0.095, *β* ^−^ = 0.316, *α* ^+^ = 49, *α* ^−^ = 1, *ω*_0_ = 0.6283 (0.1 Hz), *N* = 500, *c* = 0.02, and *ω_cut_* = 3. In all the simulations we have performed over 200 noise realizations approximated by equation *G*(*t*) and then averaged. The left column in figure 6 shows the neurons spectrum amplitude as a function of the noise intensity *σ* ^−^. As it would be expected for low noise intensities the energy transfer from the noise to the signal is not enough to achieve the synchronization and as a result the spontaneous activity dominates and no firings occur. However as the noise intensity increases firings also increase up to a maximum peak, where the mean escape rate approximately equals the signal frequency. Beyond this point, random firings can occur at different frequencies meaning that the synchronized energy transfer from the noise to the signal is destroyed and the signal is embedded in the spontaneous activity. The insert (left column, middle row) shows the well known SR inverse u-shape function, the maximum peak is found when *P* = 10 *dB*. Right column in figure 6, shows neuron's firings histograms with their correspondent time histories.

#### Crossmodal SR neuron firing condition

**Figure 7:**
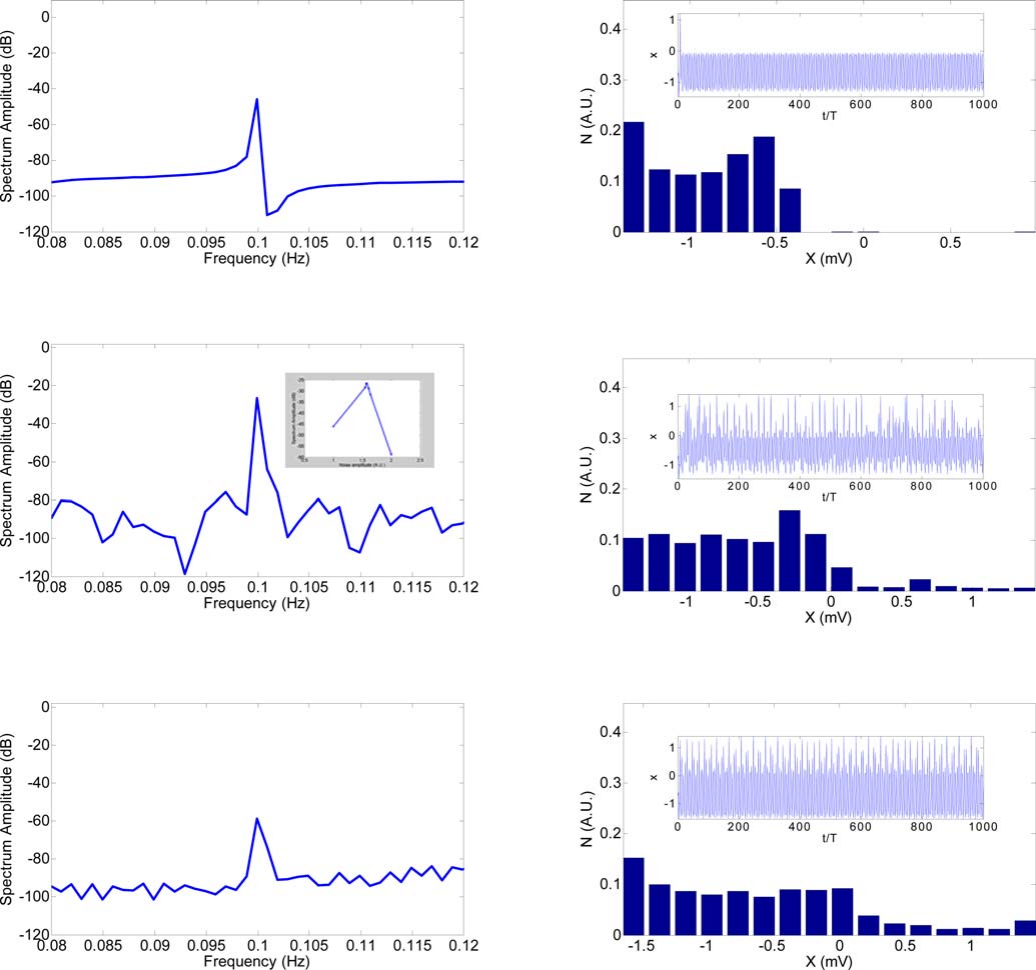
Theoretical model results for crossmodal SR. (Left column) shows the neurons' spectrum amplitude as a function of the noise intensity *σ* ^−^. The insert (left column, middle row) shows the well-known SR inverted u-shape function. The maximum peak is found when *P* = 70 *dB*. Right column shows neuronal firing histograms with their corresponding time histories. T is the signal period and N means the probability to have certain neuronal activity levels.

For crossmodal SR neurons, the signal (harmonic term in equation 1) is applied in a different region than the noise (*x*>0) and the noise is left in the same region as before (*x*≤0). In this case the inherent system perturbation is defined by parameter values *ε* ^−^>0 for *x*≤0 and *ε* ^+^>0 for *x*>0. Note that with no loss of generality, we can choose *ε* ^±^ = 1. This merely affects the choice of the parameters *γ*, *σ* and *β* in both half- planes. To achieve good neuronal time history simulations in this case *γ* ^+^≪1 because *ε* ^+^ = 1. To simplify the mathematics and the simulation time we have neglected energy losses in the half-plane *x*>0. In order to calculate the neuron's firing necessary condition, we have to take into account both homoclinic orbits Γ^−^ and Γ^+^ and parameters associated with both regions. Substituting again equation (11) and equation (10) for Γ^−^ and Γ^+^ into equation (5) then the necessary condition for the escapes to take place is
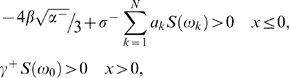
(15)but this pair of equations is equivalent to equation (14) because we are interested in the total system energy. In the unimodal case in the region *x*>0 the only contribution to the energy comes from the potential energy *V*(*x*) which is zero once it is integrated over the homoclinic loop Γ^+^. So, from equation (15) it is clear that if the maximum crossmodal SR peak is found when *P* = 70 *dB* (*σ* ^−^ has been increased seven times) then the energy loss term necessarily needs to be increased seven times as well to maintain the condition (15) similar to condition (14) where *P* = 10 *dB*. In other words, the condition for crossmodal SR to occur is that the energy transfer from the noise and the signal to the system is the same that in the unimodal SR case, meaning that the energy transfer is fixed independently of whether is unimodal or crossmodal SR. Figure 7 shows some simulations for the behavior of neuron activity with different noise amplitudes. The parameters we used are *γ* ^+^ = 0.095 (*γ* ^−^ = 0), *β* ^+^ = 0 (*β* ^−^ = 2.2), *σ* ^+^ = 0, *α* ^+^ = 49, *α* ^−^ = 1, *ω*_0_ = 0.6283 (0.1 Hz). The averaging was performed over 200 noise realizations approximated by equation G(t) with *N* = 500, *c* = 0.002, and *ω_cut_* = 3. The left column in figure 7 shows the neurons spectrum amplitude as a function the noise intensity *σ* ^−^. As expected for low noise intensities, the energy transfer from the noise to the signal is not enough to achieve the synchronization and as a result, the spontaneous activity dominates and no firings occur. However as the noise intensity increases firings also increase up to a maximum peak, where the mean escape rate approximately equals the signal frequency. Beyond this point, random firings can occur at different frequencies meaning that the synchronized energy transfer from the noise to the signal is destroyed and the signal is embedded in the spontaneous activity. The insert (left column, middle row) shows the well-known SR inverse u-shape function, the maximum peak is found when *P* = 70 *dB*. Right column in figure 7, shows neuron firing histograms with their correspondent time histories. It is evident that in this case the excitation and energy loss has augmented in the same proportion because the spontaneous activity amplitude was larger than in the unimodal case.

#### Neuron firings with harmonic signals instead of white noise excitation

One interesting prediction from this energy and frequency based model is that white noise is not needed to produce synchronized neuron firings. In equation (5) we can interchange the stochastic process *G*(*t*) for a second harmonic signal *σCos*(*ω*_1_ *t*) instead. The necessary condition for neuron firings then becomes
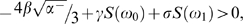
(16)From figure 5 (bottom) we observe that in order to maximize the energy transfer from the signal *σCos*(*ω*_1_ *t*) to the neuron's activity the frequency *ω*_1_ must be centered at the Melnikov scale factor *S*(*ω*)peak.

We believe this model can qualitatively explain some results that we already have presented in [14] where a series of multisensory integration interactions based on harmonic signals show similar SR-type dynamics.

### Experimental and theoretical evidence for the fixed noise energy transfer in unimodal and crossmodal SR

**Figure 8:**
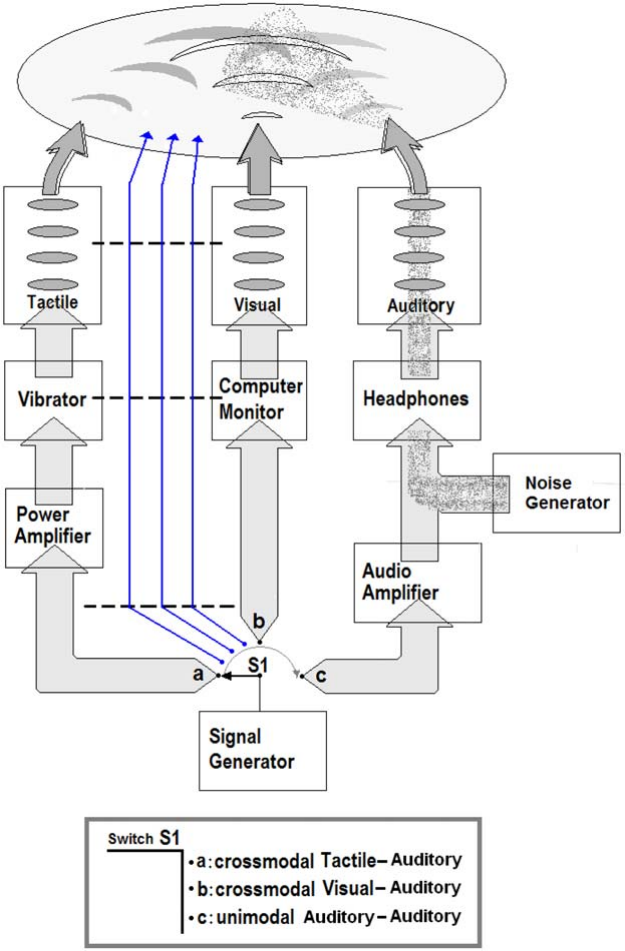
Unimodal and crossmodal SR architecture. The scheme represents the physical paths through which the signals combine in the brain.

**Figure 9:**
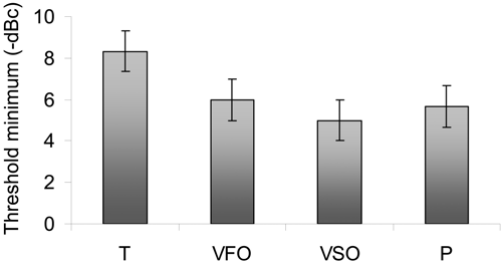
Crossmodal SR threshold minima in ceiling decibels. Shows the averaged SR minima for the four experiments discussed. For our visual experiments the minima are localized (below the noise ceiling levels) at 6±1 dBc (first order) and 5±1 dBc (second order). In the proprioception experiments the minima happened around 6±1 dBc and for the tactile experiments at 8±1 dBc.

From unimodal SR studies it can be inferred that 70 dBSPL is much louder than the noise required for auditory SR [25]–[26]. This may make the SR label we have used here problematic. However the auditory unimodal SR works in a simpler architecture than the crossmodal SR as shown in figure 8, where more neuronal networks are necessarily involved between modalities. Since the crossmodal architecture is vaster, and complex, one would expect more energy losses in such network and according with the model we have developed it is possible to have SR with these conditions. The aforementioned studies have shown that auditory unimodal SR happens between 5 dB [25] and 3–5 dB [26] below a point defined as noise threshold [26]. The noise threshold is the point where the noise hinders the signal detection and the sensitivity worsens to levels above threshold (the crossing point in the inverse u-shape curve). If we use this level as our reference instead of the SPL absolute scale (we will call this level the noise ceiling level that defines a ceiling decibel **dBc**) then we found that crossmodal SR threshold minima occur approximately in the same experimental range as the ones mentioned above. Figure 9 shows the crossmodal SR threshold minima for the four experiments presented and it is clear that for visual experiments the minima are localized at −6±1 dBc (first order) and −5±1 dBc (second order). In the proprioception experiments the minima occurs around −6±1 dBc and for tactile experiments at −8±1 dBc. The theoretical model can be used to estimate noise ceiling levels as follows: since conditions (14) and (15) are equivalent we only used condition (14). Condition (14) with parameter values *γ* ^−^ = 0.095, *β* ^−^ = 0.316, *α* ^+^ = 49, *α* ^−^ = 1, *ω*_0_ = 0.6283 (0.1 Hz), *N* = 500, *c* = 0.02, and *ω_cut_* = 3 gave a firing threshold of *σ* ^−^ = 0.147. Computer simulations gave a bigger value of *σ* ^−^ = 0.209. We increased *σ* ^−^ up to the SR peak (by analyzing the 0.1 Hz signal spectrum amplitude) and we kept increasing *σ* ^−^ until the 0.1 Hz signal spectrum amplitude was the same as at threshold (noise ceiling level). The SR peak was found at *σ* ^−^ = 0.22 and the noise ceiling level at *σ* ^−^ = 0.25. We know that the crossmodal peaks occur at approximately 70 dB therefore *σ* ^−^ = 0.22 is proportional to 70 dB. This means that the noise ceiling level would be around 79 dB. This implies that the SR peak is located at −9 dBc which is the same order of magnitude than the experimental values found for unimodal and crossmodal SR with the above parameters. These results underscore the very important fact that independently of the unimodal or crossmodal SR the energy transfer from signal plus noise is approximately fixed, which correlates with our theoretical model. Note that for measuring the noise ceiling level we have used a similar approach than the one presented in [26].

## Discussion

These results suggest a common neuronal processing mechanism for all the explored interactions. Note that we are not claiming that in these four stochastic resonances the neuronal circuits are the same. Neurons may belong to different brain regions (as depicted in figure 8) but they always follow the same physical principles. This is clear in our results because we have explored three different sensory systems and in one sensory system (visual) we have studied two different attributes corresponding to distinct mechanisms. Each system presented separate crossmodal SR characteristics but the SR minima were always in a similar range. Furthermore, there is evidence that the neuronal mechanisms involved in LM and CM detection are different and that CM involves more complex processing than LM processing [15], [16]. Nevertheless, we found similar SR characteristics. Our results strongly support the notion that there is a fundamental SR-type physical principle that underlies all sensory processing. We believe that the same principle is involved here as described in [14] where a theoretical framework is proposed to explain multisensory integration interactions based on physics dynamics. In addition, we have developed a theoretical model that can explain the experimental results herein and the results presented in [14]. The theoretical model is based in a general principle that can be summarized as follows: a subthreshold excitatory signal (entering in one sense) that is synchronous with a facilitation signal (entering in a different sense) can be increased (up to a resonant-like level) and then decreased by the energy and frequency content of the facilitation signal. As a result the sensation of the signal changes according with the excitatory signal strength. In this context, the sensitivity transitions represent the change from spontaneous activity to a firing activity in multisensory neurons. Initially the energy of their activity (supplied by the weak signals) is not enough to be detected but when the auditory noise enters the brain, it generates a general activation among multisensory neurons, modifying their original activity. In our opinion, the result is an integrated activation that promotes sensitivity transitions and the signals are then perceived. In other words, the activity created by the interaction of the excitatory signal (for example tactile) and the facilitation signal (auditory noise) at some specific energy, produces the capability for a central detection of an otherwise weak signal. We have previously shown that this principle is similar for deterministic and SR type transitions [14]. Because this multisensory facilitation process appears universal and a fundamental property of sensory/perceptual systems, we will call it the multisensory FULCRUM principle. A fulcrum is one that supplies capability for action and we believe that this best describes the fundamental principle at work in these multisensory interactions. Moreover the energy transfer from the facilitation plus the excitatory signal to the system is approximately fixed whether is unimodal or crossmodal SR, which correlates with our theoretical model as well.

From a neuroscience perspective we can hypothesize that the crossmodal SR we observed in our experiments might be associated with the simultaneous activation of multisensory neurons in different brain regions once the noise enters. For instance, in the superior colliculus (SC) there are multisensory neurons exhibiting overlapping cross-modal visual and auditory receptive fields and in the posterior parietal cortex (PPC) there are multisensory neurons exhibiting overlapping cross-modal auditory and somatosensory receptive fields [27]–[33]. Furthermore, the PPC receives proprioceptive, visual, auditory, limbic and motor inputs [27]–[33].

Since we are using auditory noise, one might argue that 70 dBSPL (clearly audible) could be judged annoying by some people (although previous crosmodal SR claims have shown that this is the effective range [11]). Indeed the threshold for sound annoyance is a complex phenomenon and there can be no single predetermined value that corresponds to it. Indeed, there are reports of high levels of annoyance for very soft sounds (e.g. 35 dBA sound of a toilet flushing from an apartment above) [34]. Annoyance is defined by the context, and 70 dB SPL white noise for a normal hearing person could easily be construed as annoying under some conditions, for example if it were perceived to affect performance in an experiment where the participant wanted to do well. Indeed, subjects were exposed to white noises from 60–95 dB SPL during the experiment, so the noise could have been construed as interfering and annoying at all of the levels used, and could have caused arousal optimal for the task at around 70 dB. From these arguments one could possibly advance the hypothesis that the crossmodal effects are due to arousal. Arousal is a physiological and psychological state of being awake and represents physiological readiness for activity. Readiness or preparedness is the state of having been made ready or prepared for use or action. We argue that this classic definition of arousal cannot account for the crossmodal facilitation results presented here and elsewhere [11], [14] for several reasons. First our experimental conditions were all randomized and our subjects naïve, which would reduce the possibility of being specifically prepared for one condition or another. Second, we have shown that we can obtain similar dynamics with deterministic signals experimentally [14] and via modeling here. Given that the deterministic facilitation signals were simultaneously paired with the detection signal (no anticipation) we can also argue that the classic definition of arousal from noise would fall short at explaining these dynamics. Further, from the model we have developed it is clear that it is not the stochastic process that defines the noise (its uncontrollable nature) that makes the synchronization-like phenomenon occur. Instead it is the energy and frequency that are contained in the noise (or a harmonic signal) and the interaction between the excitatory and facilitation signals that makes the phenomenon possible and allows the subjects to improve perception. Nonetheless the fact that the subject's perception is enhanced by SR mechanisms might change the subjects' behavior if we would ask them to do a second task in parallel with the detection task such as in behavioral SR [9]. This implies that known behavioral effects induced by noise may have their origin at a lower level. We therefore propose that SR could possibly explain properties of the Yerkes-Dodson law dynamics under certain conditions but this is beyond the scope of this work. Another argument can be made against a simple arousal interpretation of crossmodal SR. We found that the crossmodal SR effect was similar between luminance versus contrast-defined stimuli. It is well known that such stimuli require different processing levels where the contrast-defined stimuli are more complex to process [15], [16] and are differentially affected by other factors such as attention, fatigue and learning. We would therefore have expected a greater and different effect of arousal on the contrast defined stimuli but we did not find this. Rather we found very similar results and this would be difficult to account with a simple arousal explanation.

It is clear from our experimental controls, that the differences between subjects' peaks cannot be explained by criteria shifts, attention-related effects or auditory and/or visual anomalies. Thus we can conclude that these differences come from the way the individual brains process the energy (and probably the frequency) content of noise and signal. However this processing could be affected by other factors that we cannot control, for instance irregularity of the background activity at the superior colliculus, thalamic and cortical levels, etc, [11], which requires further study.

Our results suggest exciting new directions. For instance, can this or an extended theoretical framework unify the multisensory integration results and crosmodal SR? What are the possible neural substrates that could explain these interactions? How will these dynamics be mathematically represented? And what are the physical frequencies involved in each interaction? Finally, these results have obvious implications in developing methods for enhancing human performance in easy non-invasive ways. One possible application is with the elderly. As we age, we depend more and more on multisensory perception. In the presence of any one sensory deficit, such as presbyopia or presbycusis, or any age-related neurobiological alteration crossmodal SR takes on a new and important meaning. It has been recently suggested that, despite the decline in sensory processing that accompanies aging, the use of multiple sensory channels may represent an effective compensatory strategy to overcome these unisensory deficits [35].

## Materials and Methods

### General Procedure and Experimental section

**Figure 10:**
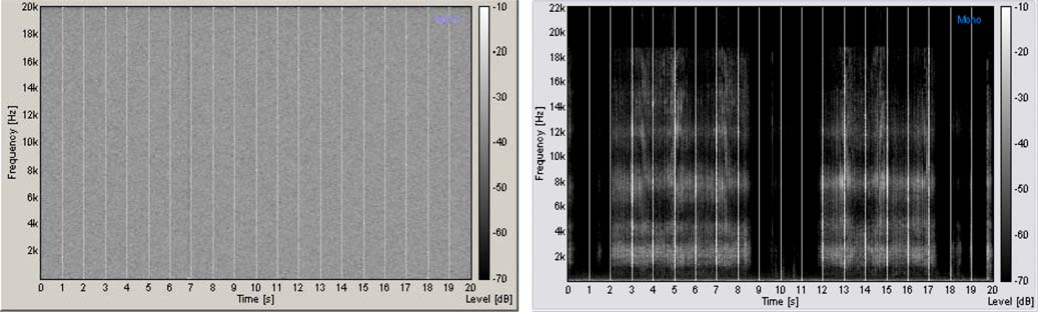
Auditory stimuli representation. Frequency-time representation of auditory noise (left) and the 3D sound (right).

This study obtained ethics approval from the CERSS (Comité d'éthique de la recherche des sciences de la santé) of Université de Montréal where all the testing took place. Informed written consent was obtained from all participants of the study. The experiments took place in a dark room for vision testing and illuminated for tactile and posture testing. The auditory stimuli were presented binaurally by means of a pair of headphones (Grado Lab SR80) plugged in an amplifier (Rolls RA62b). We used a calibrated high fidelity capacitor microphone (Behringer ECM8000) to verify the frequency response of the headphones inside an acoustically isolated chamber. A computer provided auditory white noise to the amplifier and the intensity range of the noise generator was calibrated to supply white noise with an intensity of 60 to 95 dBSPL with a systematic error of 3.5 dBSPL. The testing room sound disturbances (e.g., computer fans and low power sounds coming from outside the testing room) were recorded by using the same microphone as before and their cut-off frequency was 2.5 kHz with intensity of 50±3.5 dBSPL. Although the noise generation band and the electronic amplification bandpass are wider than the auditory spectrum, the acoustic transducer of the headphones drastically modifies the noise spectrum density because of mechanic and electric resonances. The most limiting factors at successive stages are the headphones that cannot reproduce the full white noise spectrum but still have an effective acoustic noise spectrum. Common transducers have a high cut-off frequency between 10 and 12 kHz but the human auditory noise thresholds are best for a noise spectrum between 5 and 12 kHz (ITU-R 468 noise weighting standard) partially compensating each other. The different processing stages required for the original noise to finally reach the cortex inevitably modify the original white noise spectrum. This implies that the cortex interprets only a limited noise band with a cut-off frequency of about 12 to 15 kHz (where its spectrum is attenuated) instead of a full white noise spectrum. We evaluated the subjects' hearing, from 250 Hz to 8 KHz, in A 6′×10′ double wall IAC audiometric sound suite that met the ANSI standard (Standard 3.1-1991) for permissible ambient noise levels (in one-third octave bands) for testing in free-field and under headphones. We used an audiometer (Midimate 602). All the subjects had normal hearing with a group minimum of 8±4.5 dBSPL at 1200±200 Hz. Figure 10 (left) shows a time-frequency spectrogram example of auditory noise and The figure 10 (right) shows a time-frequency spectrogram example of the 3D sound stimuli.

### Auditory-tactile

Tactile vibrations were delivered to the middle finger of the right hand of the subjects by using a VSA-3000 System (Medoc Ltd) at a frequency of 100 Hz. We have used a yes-no procedure implemented in trials with four randomly interleaved staircases (each known as 1down-1up). In each 1down-1up staircase, the stimuli amplitudes were increased by 0.3 microns until the first “yes” response. The amplitudes were then decreased by one half of the initial step until a “no” response was given. Subsequently, the direction changes according to the response: increasing for “no” and decreasing for “yes”. The step was halved at every direction change. The staircase was terminated when the step size reached 0.05 microns. The threshold was determined by taking the geometrical mean of the last seven reversals and it is given in microns. All staircases began with different stimuli amplitudes randomly selected. The time between each stimulus randomly varied between 4 s and 6 s and a short beep preceded each stimulus presentation. Up to twelve null stimuli (absence of tactile stimulus) were randomly presented in each staircase. The total number of stimuli for each staircase could not exceed 72 (including the null stimulus) or otherwise terminated. Each subject was tested twice for every auditory condition including the baseline.

### Auditory-visual

All the stimuli used in this experiment are the sum of two terms: a luminance modulation *L_LM_*(*x*, *y*) and a contrast modulation *L_CM_*(*x*, *y*) given by:
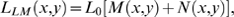
(1)

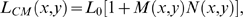
(2)where *L*_0_ represents the stimulus luminance average and the background luminance and *N*(*x*, *y*) is and external carrier function. The function *M*(*x*, *y*) is defined as:
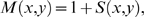
(3)where *S*(*x*, *y*) is the signal.

**Figure 11:**
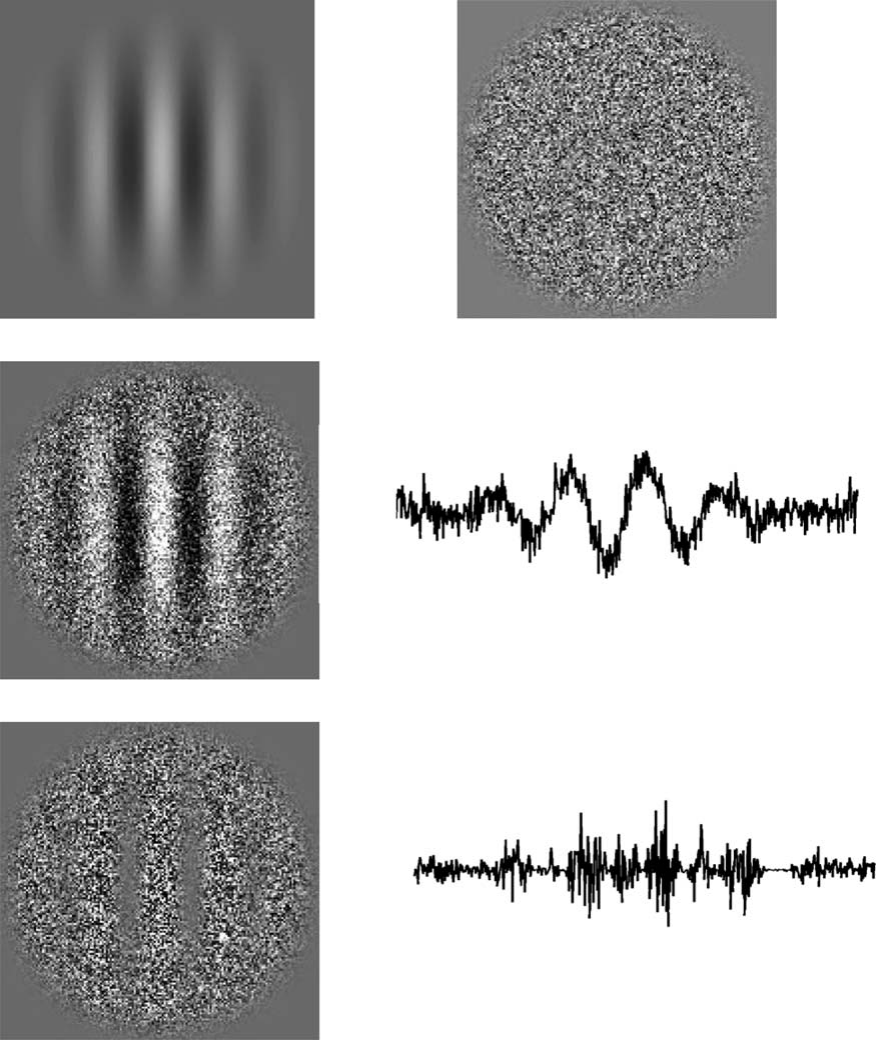
Visual stimuli representation. (Top row) Gabor patch signal (left), and carrier consisting of Gaussian noise (right). (Middle row) the spatial representation for luminance modulated (first order) stimuli. (Bottom row) the spatial representation for contrast modulated (second order) stimuli.

The signal function (*S*(*x*, *y*)) is a Gabor patch displayed in Figure. 11 (top row, left) with a center spatial frequency *f* of 1 cpd, a standard deviation *σ* of 1 deg, a phase *p* randomized at each stimulus presentation and a Michelson contrast *C* (*C_LM_* or *C_CM_* depending on the type of modulation) that varied according to the task (see below) *S*(*x*, *y*) is given by:
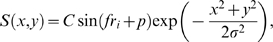
(4)where *r_i_* can be the direction *x* or *y*. The carrier function *N*(*x*, *y*), shown in Figure 11 (top row, right), generated a matrix of 320 times 320 pixels (5 times 5 deg), each element being randomly selected from a Gaussian distribution centered on 0.

In words, we define LM stimuli as the addition of an envelope (signal) with a carrier (texture) (Figure 11, middle row, left) and CM stimuli as their multiplication (Figure 11, bottom row, left). Consequently, for LM stimuli, the local luminance spatial average varies throughout the stimulus according to the envelope while the local contrast remains constant (Figure 11, middle row, right). For CM stimuli, the local luminance spatial average remains constant and the local contrast varies throughout the stimulus according to the envelope (Figure 11, bottom row, right). Therefore, because a Fourier transform can directly detect the signal frequency of LM stimuli, this type of stimulus is typically characterized as Fourier, first order, or linear. However, CM stimuli are not considered as Fourier stimuli because the signal frequency is not present in the Fourier domain. Therefore, CM stimuli are characterized to be non-Fourier, second order, or nonlinear stimuli.

The stimuli were presented using a 19 in ViewSonic E90FB .25 CRT monitor with a mean luminance of 43 cd/m^2^ and a refresh rate of 100 Hz, which was powered by a Pentium 4 computer. The 10-bit Matrox Parhelia 512 graphic card could produce 1024 gray levels that could all be presented simultaneously. The monitor was the only light source in the room. A Minolta CS100 photometer interfaced with a specific developed program calibrated the output intensity of each gun. At a viewing distance of 2.20 m, the width and height of each pixel were 1/64 deg of visual angle.

In all the conditions, a 2-alternative-forced-choice method was used: every presentation contained a carrier modulated by a signal but the Gabor patch was either horizontal or vertical. The task was to discriminate between vertical or horizontal luminance-modulated stimuli and contrast-modulated stimuli. For a given task (detection of a LM or CM signal), the signal and carrier modulation types were fixed and known to the observer. The stimuli were presented for 500 ms with stimuli intervals of the same duration. The spatial window was circular with a full contrast plateau of 4 deg width and soft edges following a Gaussian distribution with a SD of 0.25 deg. After each trial, a feedback sound indicated to the observer if his response was correct. To evaluate thresholds, a 2-down-1-up procedure was used, that is, after two consecutive correct responses the dependant variable, *C_LM_* or *C_CM_* depending on the task, was decreased by 10% and increased by the same proportion after each incorrect response. The threshold was defined as the geometric mean of the last 6 inversions (peaks) of the dependent variable values. Participants were seated at a distance of 2.20 m of a calibrated computer screen and they had to decide whether the presented grating was horizontal or vertical. We measured the LM and CM thresholds for the six auditory conditions (baseline plus five noise levels) in a random order. Five thresholds (5 separate staircases) were established for each condition and averaged.

### EMG measurement

Subjects were asked to stand with the feet one in front of the other and touching like in a tightrope position. The muscular activity was measured with a Bagnoli-2 EMG system. EMG potentials were recorded by using an active differential surface electrode placed on the right calf (gastrocnemius medial head). The EMG potentials were measured with respect to the electric potential of a neutral inactive site located away from the EMG muscle source (left pectoralis major) and we have used a 3 M Red Dot conductive electrode as the reference electrode. Both, the differential and reference electrodes were connected to an amplifier (gain of 1000) with a sampling frequency of 1000 Hz. The EMG signals were then stored out for further analysis. Every subject was tested three times for each auditory condition including the baseline in a randomized order. Every EMG measurement lasted 30 seconds with one minute rest between measurements. After the data was collected the power spectral density (PSD) of each trial was obtained and averaged for each auditory condition. The normalized power was then calculated by dividing the PSD integral (for each auditory noise condition with the corresponding PSD integral (for each baseline condition) and then averaged.
